# Soothing the emotional brain: modulation of neural activity to personal emotional stimulation by social touch

**DOI:** 10.1093/scan/nsz090

**Published:** 2019-12-10

**Authors:** Jakub Kraus, Andreas Frick, Robert Roman, Lenka Jurkovičová, Radek Mareček, Michal Mikl, Milan Brázdil, Mats Fredrikson

**Affiliations:** 1 Department of Clinical Neuroscience, Karolinska Institute, Stockholm, Sweden; 2 Faculty of Medicine, Masaryk University, Brno, Czech Republic; 3 Centre for Neuroscience, Central European Institute of Technology, Masaryk University, Brno, Czech Republic; 4 The Beijer Laboratory, Department of Neuroscience, Uppsala University, Uppsala, Sweden

**Keywords:** holding hands, anterior cingulate, anterior insula, attachment, connectivity

## Abstract

Social touch may modulate emotions, but the neurobehavioral correlates are poorly understood. Here, we investigated neural responses to a picture of a deceased close person and if neural activity and connectivity are modulated by social touch from one’s romantic partner. Using functional magnetic resonance imaging, we found altered reactivity in several brain areas including the anterior cingulate cortex (ACC) and the anterior insula in response to the personal picture compared to a picture of an unfamiliar person. Hand holding with the romantic partner, compared to being alone, reduced reactivity in the ACC and cerebellum and provided subjective comfort. To separate physical touch from the emotional effect of partner presence, we evaluated hand holding with the partner relative to a stranger and found reduced reactivity in the anterior insula. Connectivity between the anterior insula and the ACC was reduced during partner touch, and the connectivity strength was negatively related to attachment security, with higher reported partner security associated with weaker connectivity. Overall, holding hands with one’s partner attenuates reactivity in emotional brain areas and reduces between-region connectivity.

## Introduction

Viewing pictures of deceased relatives induces sadness and engages a network of brain regions including the anterior and posterior cingulate cortices, caudate nucleus, cerebellum, anterior insula, fusiform gyrus, inferior temporal gyrus and periaqueductal gray (Gündel *et al*., 2003; O’Connor *et al*., 2008), which largely overlaps with brain areas involved in processing of general emotional pictures (e.g. Geday *et al*., 2003; Fusar-Poli *et al*., 2009; Sabatinelli *et al*., 2011). The overlap probably reflects the emotional components of being reminded of the deceased person (Damasio *et al*., 2000; Gündel *et al*., 2003;”” O’Connor *et al*., 2008).

Emotion-related neural reactivity and subjective experiences can be regulated by being with other individuals, for example one’s romantic partner (e.g. Coan *et al*., 2006, 2013; Johnson *et al*., 2013). However, in comparison to the vast literature on brain effects of exposure to emotional stimuli (Fusar-Poli *et al*., 2009; Sabatinelli *et al*., 2011), there is a scarcity of studies on modulation of emotional reactions, including the effect of social touch. There is some evidence that the presence of and physical contact with one’s romantic partner reduces neural reactivity to physical threat and pain (Eisenberger *et al*., 2011; Coan *et al*., 2006, 2013; Johnson *et al*., 2013) and that this effect varies as a function of attachment style—i.e. individual differences of relating to close others while under stress (Bowlby, 1982; Carpenter & Kirkpatrick, 1996; Mikulincer *et al*., 2003; Mikulincer & Shaver, 2005). Interestingly, the insula, anterior cingulate cortex (ACC) and striatum, brain areas engaged by viewing images of deceased relatives, are also associated with adult attachment (e.g. Feldman, 2017; Laurita *et al*., 2019) and social emotion regulation (Grecucci *et al*., 2013), making them likely to be activated when seeing a picture of a deceased close person and to be regulated by tactile social contact with a romantic partner. However, empirical studies are lacking and neural correlates of partner presence during exposure to stressors qualitatively different than physical threat and pain are largely unknown.

Thus, we aimed to investigate possible effects of holding hands with a romantic partner on neural responses to personally, highly emotional visual stimulation. First, to identify brain areas that display increased activity in response to pictures of a deceased loved one, participants underwent functional magnetic resonance imaging (fMRI) during presentations of a personal picture of a deceased close person and of a standardized non-personal neutral picture. Next, we tested whether activity in brain areas engaged by the personal picture is altered when holding hands with the romantic partner. To separate the emotional effect of partner’s presence from the effect of physical contact, we contrasted holding the hand of a partner with holding the hand of a complete stranger. In addition, connectivity analyses were applied to investigate if neural ensembles are activated or deactivated together by partner support. Finally, we performed a correlation analysis of the connectivity strength and attachment style to investigate whether individual differences in attachment security relate to brain connectivity and reactivity. The findings can provide new leads in our understanding of the neurobiology of attachment and partner emotion regulation.

## Methods

### Participants

Participants were eligible for the study if they were at least 18 years of age (mean = 24; s.d. = 3.7), had an ongoing romantic relationship but no current psychiatric or neurological condition and if a close person recently died (time from death—median = 2.5 months). Participants were recruited through leaflets, online advertising and social networks. The experiment was performed in 32 participants (12 men and 20 female) and their partners. All participants and their partners signed an informed consent form. The study conformed to the Declaration of Helsinki (World Medical Association, 2013) (7th revision, 2013) and was approved by the Ethical Committee of Masaryk University.

### Procedure

Participants underwent three counterbalanced experimental conditions while being in the MR scanner—holding the partner’s hand, holding a stranger’s hand and while being alone without holding a hand, termed ‘Partner’, ‘Stranger’ and ‘Alone’ conditions, respectively. During all hand holding conditions, participants viewed a personal, emotionally loaded picture of their deceased close person (‘Personal’) and an unfamiliar other neutral picture (‘Neutral’) taken from the International Affective Picture System (Lang *et al*., 2008), in a counterbalanced order, with the first picture trial (Personal or Neutral) randomly assigned. The neutral picture was identical across all participants, while the personal picture was chosen individually by each participant and had a profound emotional meaning to him or her. Other studies report that viewing a picture of a deceased close person results in substantial sadness (Gündel *et al*., 2003). Each picture trial lasted for 5 s. and within every experimental condition (Partner, Stranger, Alone), there were five repetitions of each picture type presentation (5× personal and 5× neutral). After every 5-s picture presentation, participants were requested to perform a subsequent 35-s-long mental imagery, related to the content of the picture (data not included here). The mental imagery aided the participants in staying emotionally engaged during the task. After each imagery period, there was a 10-s-long relax phase. In the Partner and Stranger conditions, participants held hands for the duration of picture presentation, imagery and relax for all trials within that condition. All experimental conditions (Partner, Stranger and Alone) were completed at the same visit. Before the experiment, participants were presented with each picture and rated the emotional intensity on a 10-point visual analogue scale ranging from 1 (no reaction) to 10 (strong reaction). Following the experiment, we asked the participants to state which experimental condition (if any) was the most comforting to them (categorical response).

## Measures

### Questionnaires

Experiences in Close Relationship—The Relationship Structures (ECR—RS) questionnaire is designed to assess attachment-related personality characteristics (adult attachment style) in close relationships while under stress. The measure has satisfactory psychometric properties (Fraley *et al*., 2011) and has recently been used in other partner emotion-regulation studies (e.g. Krahé *et al*., 2015). In short, anxious attachment is characterized by worries that the partner will not be available in times of need and individuals with high scores on attachment avoidance are characterized to be uncomfortable and distressed with intimacy and prefer not to depend on the partner in times of need. Secure attachment is thus reflected in lower scores on both anxiety and avoidance items (Fraley *et al*., 2011).

### MRI acquisition

The MRI protocol consisted of structural and functional runs within a single session using a 3T Siemens Magnetom Prisma scanner and 64-channel head-neck coil. During the structural part, T1-weighted high-resolution data were measured using MP RAGE sequence with the following parameters: TR 2300 ms, TE 2.33 ms, TI 900 ms, FA 8°, 240 sagittal slices with isotropic voxel 1 × 1 × 1 mm and in-plane FOV 224 × 224 mm, GRAPPA with PAT factor 2. T2-weighted FLAIR images were obtained as well (192 sagittal slices with in-plane FOW 256 × 256, isotropic voxels 1 × 1 × 1 mm, GRAPPA with PAT factor 2, FA 120°, TR 6000 ms, TE 387 ms, TI 1900 ms).

Functional scans consisted of three runs based on CMRR MB-EPI (echo-planar imaging with simultaneous multislice option) with identical acquisition parameters. Because of the necessity to acquire high-quality data from all brain regions, a multi-echo fMRI protocol was implemented using three echoes at 15, 33 and 52 ms. Each run consisted of 630 scans with TR 800 ms, 60 axial slices with in-plane FOV 200 × 170 mm, acquisition matrix 80 × 68, isotropic voxels 2.5 × 2.5 × 2.5 mm, pixel bandwidth 2405 Hz, FA 26°, GRAPPA with PAT factor 2, MB factor 6, phase encoding direction = AP.

### MRI data processing

All MRI data were converted to NIFTI format and processed in SPM12, build 6225 (Wellcome Trust Centre for Neuroimaging at University College London (UCL), UK. http://www.fil.ion.ucl.ac.uk/spm/) running under Matlab 8.4. R2014b. Functional MRI data were processed as follows: fMRI data from all echoes were realigned to the first scan and the first echo to correct any voxel misplacement caused by motion. Composite multi-echo data were calculated from individual echoes voxel-wise as a weighted average (Poser *et al*., 2006) according to contrast-to-noise ratio using individual tSNR values and echo times TE using the formula ${Yc}_i=\frac{\sum_{\mathrm{e}}{Y\mathrm{s}}_{\mathrm{e},i}\ast{\mathrm{tSNR}}_{\mathrm{e},i}\ast{\mathrm{TE}}_{\mathrm{e}}}{\sum_{\mathrm{e}}{\mathrm{tSNR}}_{\mathrm{e},i}\ast{\mathrm{TE}}_{\mathrm{e}}}$, where *i* stands for index of individual voxel, *e* stands for index of individual echo, *Y*c*_i_* represents the composite BOLD data (time-series) in voxel *I*, *Y*s_e,*I*_ represents the motion-corrected BOLD signal (time-series), tSNR represents temporal signal-to-noise ratio calculated for each voxel and echo as a mean of BOLD signal divided by its standard deviation (Krüger & Glover, 2001) and TE_e_ is echo time for specific echo. The composite data were transformed into the standard stereotactic MNI space and spatially smoothed with Gaussian filter (FWHM = 5 mm). Voxel size was left original (2.5 × 2.5 × 2.5 mm isotropic). T1-weighted anatomical high-resolution data were spatially normalized into the MNI space.

The general linear model as implemented in SPM12 was used for statistical analysis of fMRI data. The contrasts of the difference between personal and neutral picture presentation (personal>neutral) were included as regressors in three first level models—separately for Alone, Stranger and Partner conditions (together with realignment parameters from the respective motion correction step). These contrasts were then entered into a group-level analysis, separately for the Alone condition to identify brain areas with reactivity differences to personal as compared to neutral picture presentations. Subsequently, we performed a two-way Condition (Alone, Stranger and Partner) × Stimulus (personal and neutral pictures) interaction analysis. This allowed us to test potential differences in brain reactivity between personal and neutral picture presentations between experimental conditions. Specifically, we predicted higher brain reactivity to personal (>neutral) pictures in the Alone (>Partner), Alone (>Stranger) and Stranger (>Partner) comparisons.

The statistical threshold for significance for all analyses was set to *P* < 0.05 family-wise-error-corrected (FWE) at a cluster level with the voxel level cluster-defining threshold *P* < 0.001 uncorrected. Starting with the personal (>neutral) picture presentation in the Alone condition, a priori anatomical regions of interest (ROIs) were chosen, based on earlier findings (Geday *et al*., 2003; Gündel *et al*., 2003; Etkin *et al*., 2011; Yoon *et al*., 2007; O’Connor *et al*., 2008; Kraus *et al*., 2018). These included fusiform and inferior occipital gyrus, anterior insula, the amygdala, anterior and posterior cingulate cortices, caudate, medial orbital cortex and periaqueductal gray (PAG), regions that were previously associated with increased reactivity to emotionally important visual cues (e.g. Geday *et al*., 2003), to personal pictures (Gündel *et al*., 2003; O’Connor *et al*., 2008), to emotionally valenced pictures (e.g. Yoon *et al*., 2007, Kraus *et al*., 2018) and various other negative emotional stimuli (e.g. Etkin *et al*., 2011). All ROIs except of the PAG, were defined using the Automated Anatomical Labeling (AAL) library from the Wake Forest University Pickatlas (Maldjian *et al*., 2003). The PAG mask is not included in the AAL, and therefore, we used the emotion-related peak coordinates [1,−29,−11] from Linnman *et al.*’s 2012) review paper and created a 2-mm sphere around it. For the anterior insula ROI, similarly to other studies investigating this region (e.g. Way *et al*., 2009), the whole insula mask from the AAL was used and then restricted with a caudal boundary of *y* = 4 to delineate the anterior and posterior insula. The AAL atlas was also used to map voxel coordinates to brain regions. All coordinates are reported in MNI standard space. For the Alone vs. Partner, Alone vs. Stranger and Stranger vs. Partner comparisons, we used the ROIs that differentiated personal from non-personal pictorial stimulation in the Alone condition.

Lastly, we performed connectivity analyses by employing the Generalized Form of Context-Dependent Psychophysiological Interactions (gPPI) (McLaren *et al*., 2012). We tested whether there are any potential neural ensembles involved that are specifically affected by the partner support, using the area that showed differentiation between stranger and partner hand holding as the seed region. Specifically, we used the voxel displaying the maximum difference between the Partner and the Stranger conditions derived from the significant anterior insula cluster as the seed region and performed whole brain functional connectivity analysis.

### Behavioral and demographic data

All behavioral and demographic data were analyzed using IBM SPSS Statistics 22 software.

## Results

The personal pictorial stimulation resulted in significantly higher subjective emotional arousal (*M* = 7, SEM = 0.39) than the neutral visual stimulation (*M* = 2.4, SEM = 0.30), *t*(30) = 10.5, *P* < 0.001, *d* = 2. A majority of the participants (72%) reported that they were most comforted when holding their partner’s hand, as compared to holding a stranger’s hand or when being alone χ^2^(2) = 22.6, *P* < 0.001.

### Neural responses

#### Alone

Reactivity was higher to the personal than the neutral picture in the Alone condition within our a priori ROIs bilaterally in the inferior occipital gyrus, fusiform gyrus, PAG, left anterior insula, right medial orbital cortex, right caudate and right ACC (Table 1), but not in the amygdala or the posterior cingulate. Additional exploratory whole-brain analyses showed enhanced reactivity bilaterally in the inferior frontal gyrus, left superior medial frontal cortex, left cerebellum and left precuneus. No brain regions displayed the opposite pattern with lower reactivity to personal than neutral pictures. For the subsequent Alone vs. Partner, Alone vs. Stranger and Stranger vs. Partner analyses, we used brain areas demonstrating reactivity differences between the personal and non-personal pictures in the Alone condition, as these regions differentiate between emotional and neutral slides.

**Table 1:** Summary of significant main effects of Picture type (personal emotional versus non-personal neutral picture) and two-way interactions between Picture type and the type of company (Alone vs. Stranger, Alone vs. Partner as well as Stanger vs. Partner). Significant effects indicate higher reactivity in brain regions when being alone, when being alone as compared to hand holding with partner, when being alone as compared to holding a stranger’s hand and for holding a stranger as compared to holding a partner, during viewing of personal emotional as compared to non-personal neutral pictures

	**Z**	**Cluster *P* value (fwe)** ^a^	**Volume** ^b^	***x***	***y***	***z*** ^c^
**Reactivity**						
**Alone**						
Fusiform gyrus, left	5.5	<0.0001	2546	−40	−55	−18
Fusiform gyrus, right	5.1	<0.0001	1609	38	−60	−15
Inferior occipital gyrus, right	5.1	<0.0001	1781	38	−85	−10
Superior medial frontal cortex, left	4.8	<0.0001	4000	−7	53	25
Inferior frontal gyrus, left	4.8	0.013	593	−42	23	−15
Caudate, right	4.5	0.008	265	16	3	18
Angular gyrus, left	4.5	<0.0001	3843	−52	−57	25
Medial orbital cortex, right	4.5	<0.0001	859	6	56	−15
Inferior occipital gyrus, left	4.3	<0.0001	1031	−32	−92	−8
Fusiform gyrus, right	4.2	0.029	187	43	−60	−15
Anterior insula, left	4.1	0.005	468	−35	21	−8
Inferior frontal gyrus, right	4.1	0.006	1125	41	28	−15
Cerebellum, left	4.1	0.007	1093	−2	52	−35
Precuneus, left	4	0.01	1015	−5	−57	33
Periaqueductal gray	3.8	0.031	109	3	−30	−10
Anterior cingulate, right	3.7	0.034	125	6	51	13
**Alone > Partner**						
Anterior cingulate, right	3.5	0.037	109	8	23	20
Cerebellum, left	3.5	0.032	78	−10	−50	−38
**Alone > Stranger**						
Caudate, right	4.3	0.045	78	13	13	13
**Stranger > Partner**						
Anterior insula, left	3.9	0.04	109	−37	6	−10

a ^a^Cluster-defining threshold *P* < 0.001

b ^b^Volume in cubic millimeters rounded to the nearest decimal, voxel size isotropic 2.5 mm

c ^c^Peak voxel coordinates in MNI space

#### Alone>Partner

We found a significant two-way interaction with higher reactivity to personal than neutral pictures when being alone than with the partner in the right ACC and left cerebellum, supporting that holding partner’s hand attenuates emotion-related neural reactivity (Table 1). Additionally, using a more lenient statistical threshold (*P* < 0.001 uncorrected without a cluster threshold), we detected a cluster in the anterior insula extending into the inferior frontal gyrus also demonstrating decreased brain reactivity as a function of partner’s presence. We did not find higher reactivity when being with the partner than when being alone.

**Fig. 1:**
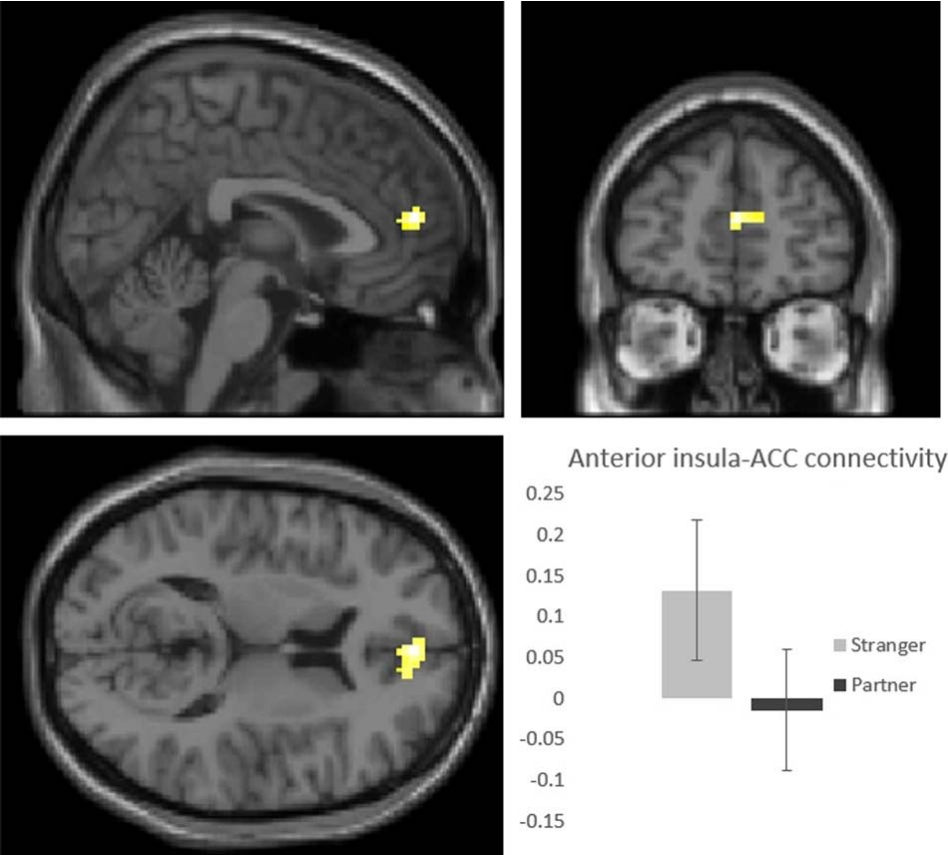
Anterior insular connectivity when holding hands with a stranger as compared to the partner for the contrast Personal > Neutral pictures. The left anterior insula showed greater connectivity with the anterior cingulate cortex during the Stranger than Partner condition (left, sagittal plane at *y* = 51; right, coronal plane at *x* = 3; bottom, axial plane at *z* = 13; pFWE = 0.004, cluster-level). The graph displays extracted measures of connectivity between anterior insula (seed) and anterior cingulate, reflecting increased connectivity during personal as compared to neutral pictures. Note that the error bars (SEM) reflect between and not within-subject variability and do not contribute to the statistical analysis of the condition effect.

#### Alone>Stranger

There was a significant two-way interaction in the right caudate, with higher reactivity during personal than neutral pictures in the Alone, as compared to the Stranger condition (Table 1), supporting attenuation of brain reactivity as a function of holding a stranger’s hand. Reactivity was not increased in any brain areas when being accompanied by a Stranger as compared to when being Alone.

#### Stranger>Partner

A cluster within the left anterior insula differentiated between holding a partner’s and a stranger’s hand, with lower reactivity to personal than neutral pictures, in the Partner than in the Stranger condition (Table 1). This differentiation indicates an additional attenuating effect of partner’s support over the support given by the stranger. Reactivity was not lower in any brain areas during the Stranger as compared to the Partner condition.

### Connectivity analyses

We investigated anterior insula connectivity using the voxel with the maximum difference in the Stranger>Partner comparison. This was chosen because it showed sensitivity to experimental perturbations and successfully differentiated between stranger and the partner presence. The gPPI whole-brain analysis revealed diminished connectivity between the left anterior insula seed and an area in the right ACC when holding hands with the partner as compared to with a stranger for personal > neutral pictures, (*x*, *y*, *z* [3, 51, 13], *Z* = 4.17, pFWE = 0.004 cluster-level, 844 mm^3^) (see Figure 1). In the same analysis, there was reduced connectivity between the left anterior insula seed and the contralateral anterior insula region (*x*, *y*, *z* [46, 18, −8], *Z* = 3.98, pFWE = 0.002 cluster-level, 984 mm^3^) as a function of partner presence. No other significant effects emerged.

**Fig. 2:**
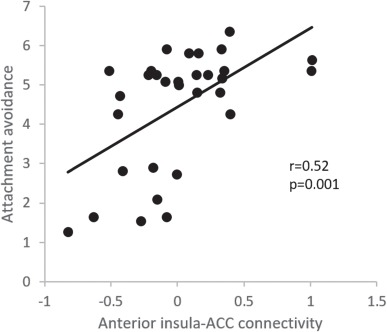
Correlation between connectivity estimates reflecting the strength of the anterior insula and ACC connectivity and degree of attachment avoidance during partner’s hand holding. Spearman’s correlation coefficient and the *P* value are displayed in the plot. Lower attachment avoidance scores reflect more secure bonding and is associated with lowered connectivity.

We investigated the role of relationship quality on the insula-ACC covariation by correlating measures of attachment security with connectivity measures (Figure 2). Even though there was no significant connectivity in the partner condition at the group level, it might be argued that the substantial variability observed might reflect variations in attachment style. This was true, as relatively higher anterior insular-ACC connectivity was related to less secure attachment (*r* = 0.52, *P* = 0.001 for avoidance and *r* = 0.33, *P* = 0.037 for anxiety). Note that higher numbers reflect less secure attachment. No correlations were observed when holding hands with a stranger (*r* = −0.06, *P* = 0.38 for avoidance and *r* = 0.08, *P* = 0.34 for anxiety). No correlations emerged between attachment security and reactivity estimates in the anterior insula or the ACC, neither in the Partner nor in the Stranger condition, or when these two conditions were compared (all *r*’s < 0.18, all *P* values >0.2). Additionally, there were no correlations between attachment security and the personal picture arousal ratings (*r* = 0.09, *P* = 0.67 for avoidance; *r* = 0.05, *P* = 0.82 for anxiety) or with neutral picture arousal ratings (*r* = −0.10, *P* = 0.63 for avoidance; *r* = −0.30, *P* = 0.14 for anxiety).

## Discussion

We investigated the modulating effect of holding hands with one’s romantic partner on neural responses to personal emotional visual stimulation. First, consistent with research on brain reactivity to emotional and personal pictures (e.g. Geday *et al*., 2003; Gündel *et al*., 2003; O’Connor *et al*., 2008; Fusar-Poli *et al*., 2009; Etkin *et al*., 2011), we demonstrated enhanced reactivity in specific brain regions including the ACC and the anterior insula to highly emotional personal pictorial stimulation. Then, we showed that activation in the ACC and cerebellum is attenuated by holding hands with the partner and that holding hands with a stranger reduced nucleus caudate activity. Also, participants reported subjective comforting from the partner’s presence. Next, we differentiated the effect of the partner’s hand holding, which has an emotional value, from holding a stranger’s hand, and found attenuated activity in the anterior insula and also diminished anterior insula-ACC connectivity, suggesting that partner presence modulates emotion-related neural activity both in single brain areas and affect neural ensemble relations. Reduced connectivity during partner presence might reflect a relatively relaxed brain as panic and fearful states induce a covariation of brain activity over multiple brain areas (e.g. Wik *et al*., 1997). In addition, with increasing attachment security, the coupling between the insula and ACC was decreased while holding hands with the partner. This supports that modulation of emotion-related neural activity depends on the perceived relation to one’s partner. It is important to note however that there was no significant connectivity in the partner condition at the group level, but that the substantial individual variability in connectivity in part can be accounted for by variations in attachment style.

Our findings of increased reactivity of the ACC, anterior insula and PAG to personal pictorial representation of a deceased close person are in line with research using personalized stimulation materials (e.g. Gündel *et al*., 2003; O’Connor *et al*., 2008) and also with research employing generic emotional visual stimulation (e.g. Fusar-Poli *et al*., 2009). Moreover, both physical (Rainville, 2002) and social pain (Rainville, 2002; Eisenberger *et al*., 2003, 2011) involve the ACC, anterior insula and PAG—regions here found to be reactive to the personal emotional pictures when being alone. This is consistent with the thought that emotional pain and discomfort is being elicited by the reminders of a particular close deceased person (e.g. O’Connor *et al*., 2008). Specifically, activity in the ACC is most likely related to emotion expression (Medford & Critchley, 2010; Dejean *et al*., 2015), whereas anterior insula, which was reduced by partner contact and had a functional connectivity with the ACC, may involve interoception and awareness, particularly in response to emotionally loaded material (Zaki *et al*., 2012; Craig, 2009).

Furthermore, our results are in accordance with studies showing attenuation of threat-related brain reactivity as a function of partner’s hand holding and support (Coan *et al*., 2006, 2013; Johnson *et al*., 2013). Because hand holding with the partner reduced anterior insula reactivity and connectivity with the ACC, we suggest that partner involvement might reduce insula activity and that insula-ACC connectivity is lower in the partner than the stranger condition because ACC insula covariation is obscured as ACC activity remained unaltered by partner presence. If the insula effect is primary, then the connectivity between insula and the ACC would imply reductions in the ACC along with insula attenuations. This was not the case as only insula, but not ACC activity, was attenuated as a function of partner presence. Also, the connectivity strength, but not reactivity differences, correlated with measures of attachment suggesting that the two processes, connectivity and reactivity, in part are independent. The coupling pattern, paired with the fact that partner presence induced comfort, suggests that subjective emotional experiences at least in part relate to the insula-ACC coupling and not just activity in each node. Indeed, there is a wealth of evidence suggesting that the ACC and anterior insular cortices have a close functional relationship crucial for generating subjective feelings (Medford & Critchley, 2010). Studies show altered subjective experiences in response to the personal pictorial material (Gündel *et al*., 2003) and also to the partner hand holding as reported in our study and in research of others (e.g. Coan *et al*., 2006). In addition, the positive correlative pattern between the anterior insula-ACC connectivity and attachment insecurity (avoidance and anxiety) suggests that the quality of emotional relating to one’s partner is an important element in attenuating this coupling, consistent with recent reviews (e.g. Laurita *et al*., 2019) and similar findings of others (Carpenter & Kirkpatrick, 1996; Coan *et al*., 2006; Eisenberger *et al*., 2011; Krahé *et al*., 2015). Eisenberger *et al.* (2011) for example found that in participants with secure attachment style, viewing partner pictures while receiving painful stimulation was associated with reductions in self-reported pain ratings concomitant with reductions in both ACC and anterior insula activity. The present paper replicates and extends previous findings of attenuating effects of hand holding on brain reactivity (e.g. Coan *et al*., 2006), by complementing these findings with the use of qualitatively different distressing stimuli and by demonstrating anterior insula-ACC connectivity and its association to attachment style. Interestingly, however, we did not find a relation between reactivity within the ACC or the anterior insula and partner bonding quality, suggesting that trust modulates connectivity rather than reactivity.

There are several limitations. First, we did not perform assessments of subjective sadness to the pictures, precluding us from performing analyses of how neural responses relate to subjective experience of low mood and grief, as we assessed only emotional arousal. Second, the personal picture was for obvious reasons not standardized and, for each participant, resulted in different proportions of facial and scenic components, possibly introducing enhanced variability in the response to the personal pictures. This would most likely act to reduce and not enhance statistical differences between the two types of pictures. Third, we did not collect subjective ratings for the personal and neutral pictures separately for holding- and not holding the hands, preventing us from assessing the interaction between the type of picture and the hand holding condition.

In conclusion, we demonstrated neurobehavioral correlates of social closeness and support. Pictures of a deceased significant other, as compared to the unfamiliar one, induced distinct neural reactivity in a priori predicted emotional brain areas including the anterior cingulate and the anterior insula. We then showed that holding the hand of one’s partner attenuated reactivity within and connectivity between regions. The insula-ACC connectivity was associated with attachment style, such that more securely attached individuals displayed attenuated connectivity with the partner present. Collectively, our findings are consistent with the notion that partner presence regulates subjective negative affect through a neural network including the anterior insula and the ACC.

## Funding

This work was supported by European Regional Development Fund-Project; National Infrastructure for Biological and Medical Imaging [CZ.02.1.01/0.0/0.0/16_013/0001775]; The Swedish Brain Foundation [F2014-0151 to M.F., FO2018-0018 to A.F.]; The Swedish Research Council [2013-02825 to M.F., 2017-01674 to A.F.]; The Swedish Foundation for Humanities and Social Science Council [P17-0256 to A.F.]; and the Kjell and Märta Beijer Foundation [A.F.].
